# The impact of humidity on the functional response of *Blattisocius Mali* (Acari: Blattisociidae) preying on the acarid mite *Tyrophagus putrescentiae*

**DOI:** 10.1038/s41598-024-78997-w

**Published:** 2024-11-14

**Authors:** Manoj Kumar Jena, Katarzyna Michalska, Marcin Studnicki

**Affiliations:** 1 Department of Plant Protection, Institute of Horticultural Sciences, Warsaw University of Life Sciences, Nowoursynowska 159, 02-776 Warsaw, Poland; 2 Department of Biometry, Institute of Agriculture, Warsaw University of Life Sciences, Nowoursynowska 159, 02-776 Warsaw, Poland

**Keywords:** Handling time, Instantaneous search rate, Mesostigmata, Predation, Soil mite, Ecology, Zoology

## Abstract

Humidity influences the life table parameters and foraging behaviours of various terrestrial arthropods. The soil mite, *Blattisocius mali* Oudemans is a potential biological control agent of some acarid mites, moths, and nematodes. In the current study, we investigated the functional response of *B. mali* preying on the eggs of the mould mite *Tyrophagus putrescentiae* Schrank (Acari: Acaridae) at different humidity levels between 33% and 92%. To determine the type of functional response, we used logistic regression and a generalized functional response equation suggested by Real. The functional response parameters were estimated using models proposed by Hassell and Cabello et al. *Blattisocius mali* exhibited Type II functional response at 33% and Type III at other tested humidities (52%, 72%, 82%, and 92%). The potential for prey mortality (α) was the highest, i.e., 0.05923, and the handling time was the shortest, i.e., 0.00463 day, at 92% humidity, indicating the highest efficiency of *B. mali* at this humidity. Our findings revealed that *B. mali* was more efficient at higher humidity levels as compared to lower humidity levels. Humidity affected the predation rate and might have played an important role in stabilizing the predator–prey system by shifting the functional response with humidity.

## Introduction

The concentration of carbon dioxide in the atmosphere is steadily increasing, leading to a rise in global atmospheric temperature by 2.0–4.5 °C by the year 2100. This rise in temperature results in the modification of precipitation patterns^1^. The warming and alterations in precipitation can directly impact soil temperature, moisture^2,3^, and humidity^4^. Humidity in turn can constrain the growth and population dynamics of various microarthropods at optimal temperatures, especially if the animal expends energy to balance hydration at the expense of reproduction^5^. Humidity plays a significant role in determining the activity, distribution patterns, and species richness of terrestrial arthropods^6^. Terrestrial arthropods include mites are particularly susceptible to water loss due to their high surface-to-volume ratio^7^. Fluctuations in humidity can cause biochemical, physiological, and behavioural changes in arthropods^6,8^ including their functional responses.

The functional response is an important aspect of predator-prey or parasitoid-host interaction and is crucial for the dynamics of populations and communities of animals^9–13^. It represents the relationship between the number of prey or hosts successfully attacked by a predator or parasitoid and the density of prey in the environment. There are four types of functional responses: Type I, where the consumption rate increases linearly until reaching a constant plateau; Type II, where the consumption rate approaches the asymptote in a hyperbolic manner as the prey density increases; Type III, where the consumption rate initially increases until reaching the inflection point of a sigmoid curve, followed by a decrease in the proportion of prey killed; and less commonly observed, Type IV, known as the domed type, where the efficiency of predation decreases instead of increasing at certain prey densities^10,12,14,15^. Among insect and mite predators, all types of functional responses have been documented^16–22^. However, as Hassell^23^ pointed out, the occurrence of Type III may be underestimated under laboratory conditions, and it may be a more common response type than previously believed.

The effectiveness of a predator can be assessed by examining the parameters of functional response, which include the predator’s instantaneous attack rate, the handling time^12^, and the predator’s potential for prey mortality^24^. Natural enemies that have high attack rates and short handling times are considered to be the most successful agents for biological control^17,25^. On the other hand, if a predator is highly efficient and causes significant mortality while reducing prey survival, the potential for causing mortality will be high, and vice-versa^24^. The type and parameters of functional response in invertebrate predators can be affected by various factors, such as temperature, prey, and predator types, predator age, voracity, hunger level, etc^26–28^.

Studies have indicated that rising temperatures have the potential to alter the functional response pattern from Type II to Type III^20–22^ and from Type III to Type II^29–31^. However, there are instances where the functional response type remained unchanged under temperature variations^32–35^. Furthermore, shifts from Type III to Type II, induced by warming, may result in the destabilization of predator-prey interactions and the potential extinction of prey species under highly unfavourable conditions^32^. All these diverse findings suggest that temperature may affect different predator-prey systems differently, depending on their sensitivity to varying temperature levels. Likewise, humidity may affect predator-prey interactions differently.

The mould mite *Tyrophagus putrescentiae* Schrank (Acari: Acaridae) is an omnivorous acarid mite, common in-house dust, soil with decomposed plant material, and vertebrate nests. It is also a pest of various stored food products and crop plants such as cucumber, gerbera, or bulbs of many ornamental plants^36,37^ and the heavy reliance on chemical pesticides for its control raises environmental and health concerns^38^. To combat these problems, low-risk methods, such as biological control have been considered as a possible alternative to chemical pesticides. Production of natural enemies and their utilization in pest control is one of the most important strategies in Integrated Pest Management programmes^39^.

This study aimed to examine the impact of five levels of relative humidity on the functional response of the predatory mite *Blattisocius mali* Oudemans towards its prey *T. putrescentiae*. *Blattisocius mali*, a type of soil mite, has garnered attention recently due to its potential to control acarid mites, nematodes, and moth pests. It belongs to the family Blattisociidae, in which several species i.e. *B. dentriticus* Berlese, *B. tarsalis* Berlese, and *B. keegani* Fox, have been reported to be the potential biocontrol agents of stored food pests including the mold mite, *T. putrescentiae*^40^. By feeding on a mixture of mould mite eggs and larvae, the life table parameters of *B. mali* were much higher than those of *B. dentriticus*, also examined in this respect^41^. Apart from acarid mites, *B. mali* has been reported to be a potentially effective predator of nematodes^42^, the eggs of moths^43,44^ as well as phytophagous insects such as thrips and mites such as spider mites^45^. Moreover, recent findings revealed that *B. mali* not only could disperse using drosophilids but also feed on them during transportation and after disembarkment, also prey upon their eggs and larvae^45–47^.

To date, there is no report on the influence of humidity on the predation of soil mites towards different prey densities. Among various climate factors, moisture seems to have the most significant impact on soil organisms, particularly those occupying higher trophic levels such as predatory mites or omnivorous nematodes. These organisms have been observed to significantly increase in abundance in response to available soil moisture^48^. As humidity has been shown to significantly affect the survival and speed of development of other *Blattisocius* species, i.e., *B. dendriticus* and *B. keegani*^49,50^, we expected that humidity might be a key factor influencing the dynamics of *B. mali* and *T. putrescentiae* interactions and stability of that system. In this paper, we pose a question on to what extent the possible deleterious effect of extreme values of humidity may affect the behaviour of a predatory mite and this way also the functioning of the whole predator-prey system. To determine the changes in the functional response of *B. mali* solely with humidity, we selected the immobile egg stage as a prey, since, the mobile stages, i.e., larva, protonymph, deutonymph, and adult, of the *T. putrescentiae* might change the behaviour with changing moisture, and additionally affect predator’s response. Apart from classical parameters of functional responses, instantaneous attack rate, and handling time^12^, we also compared another parameter, the potential of mortality (α) proposed by Cabello et al.^24^ to provide a more comprehensive biological interpretation of predator’s behaviour exhibiting Type III functional response.

## Results

**Fig. 1:**
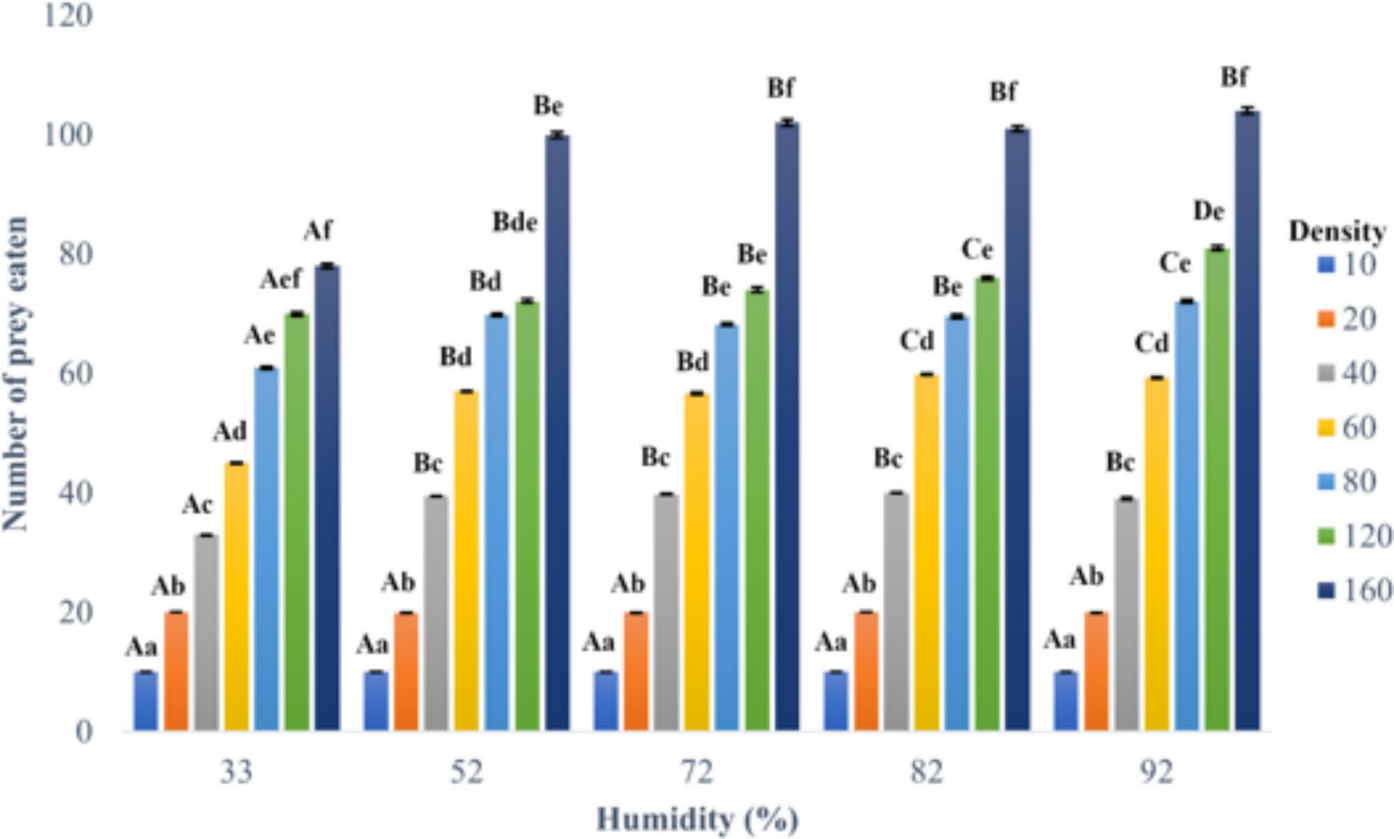
The effect of five humidity levels and seven densities of *Tyrophagus putrescentiae* eggs on the mean number (± 95% CI) of the *T. putrescentiae* eggs eaten by *Blattisocius mali.* Different lowercase and uppercase letters indicate significant differences between means (*P* < 0.05) for various prey densities within each humidity level or among different humidity levels, respectively.

The statistical analysis revealed a significant effect of both humidity (χ^2^ = 1627.23; *df* = 4; *P* < 0.001) and the density of *T. putrescentiae* eggs offered (χ^2^ = 829.38; *df* = 6; *P* < 0.001) on the mean number of eggs eaten by *B. mali*. Additionally, the interaction between humidity and density was found to be significant (χ^2^ = 829.38; *df* = 6; *P* < 0.001), implying that the mean number of eggs eaten by the predator depended not only on the specific level of humidity but also on the density of the prey. At low prey densities of 10 and 20 eggs, the mean numbers of eggs eaten by *B. mali* females were similar at tested humidity levels (Fig. 1). However, in other prey densities, the predators ate significantly fewer eggs at the lowest humidity of 33% compared to higher humidity levels. While there was no significant difference in the number of prey consumed within the humidity range of 52–92% for densities of 40 and 160 eggs, there was a noticeable trend of increasing consumption rate with higher humidity levels for other prey densities.

Logistic regression between the initial egg densities offered and the proportion of eggs eaten revealed that the linear coefficients were positive and the quadratic coefficients were negative at the levels of humidity, 52%, 72%, 82%, and 92%; indicating a Type III functional response. In contrast, both the linear and quadratic coefficients were positive at a humidity level of 33%, making it difficult to determine the exact type of functional response at this level (Table 1). Therefore, the additional method proposed by Real^51^ was also used to clarify the type of functional response which showed that the value of ‘q’ was not significantly different from zero and T_h_ was greater than zero, at a humidity of 33%; indicating a Type II response. However, at humidity levels of 52%, 72%, 82%, and 92%, both q and T_h_ were found to be greater than zero, indicating a Type III functional response (Table 2).

**Fig. 2:**
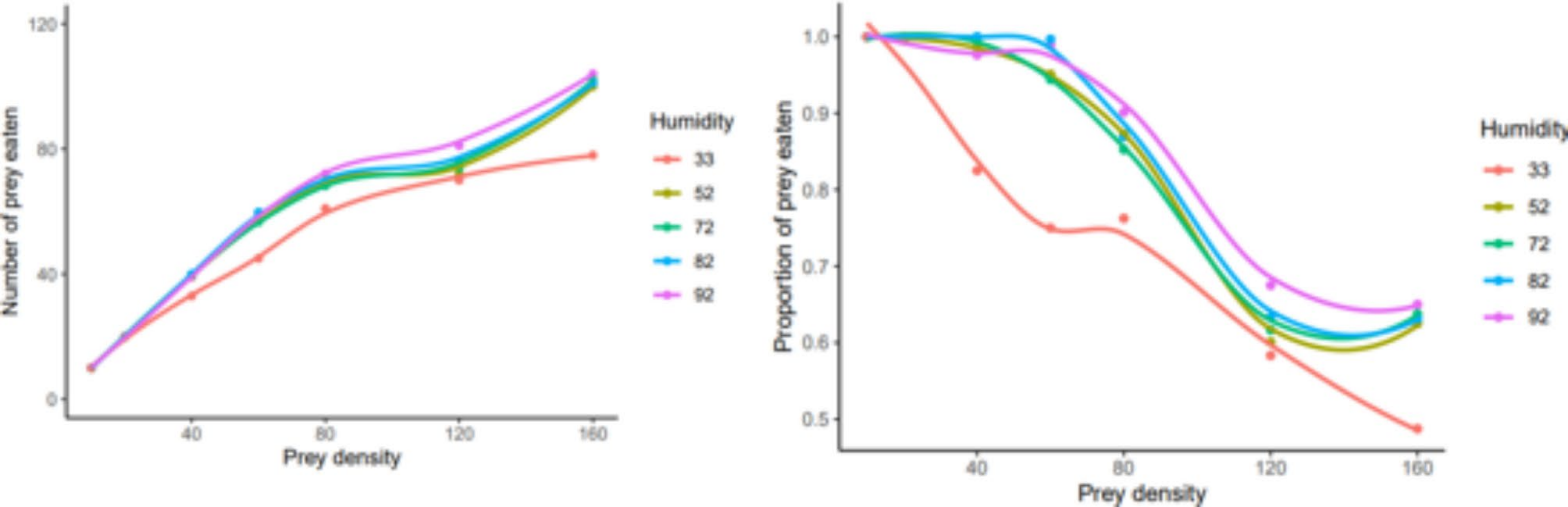
The number and proportion of the *Tyrophagus putrescentiae* eggs eaten by *Blattisocius mali* at five levels of humidity and seven prey densities.

**Fig. 3:**
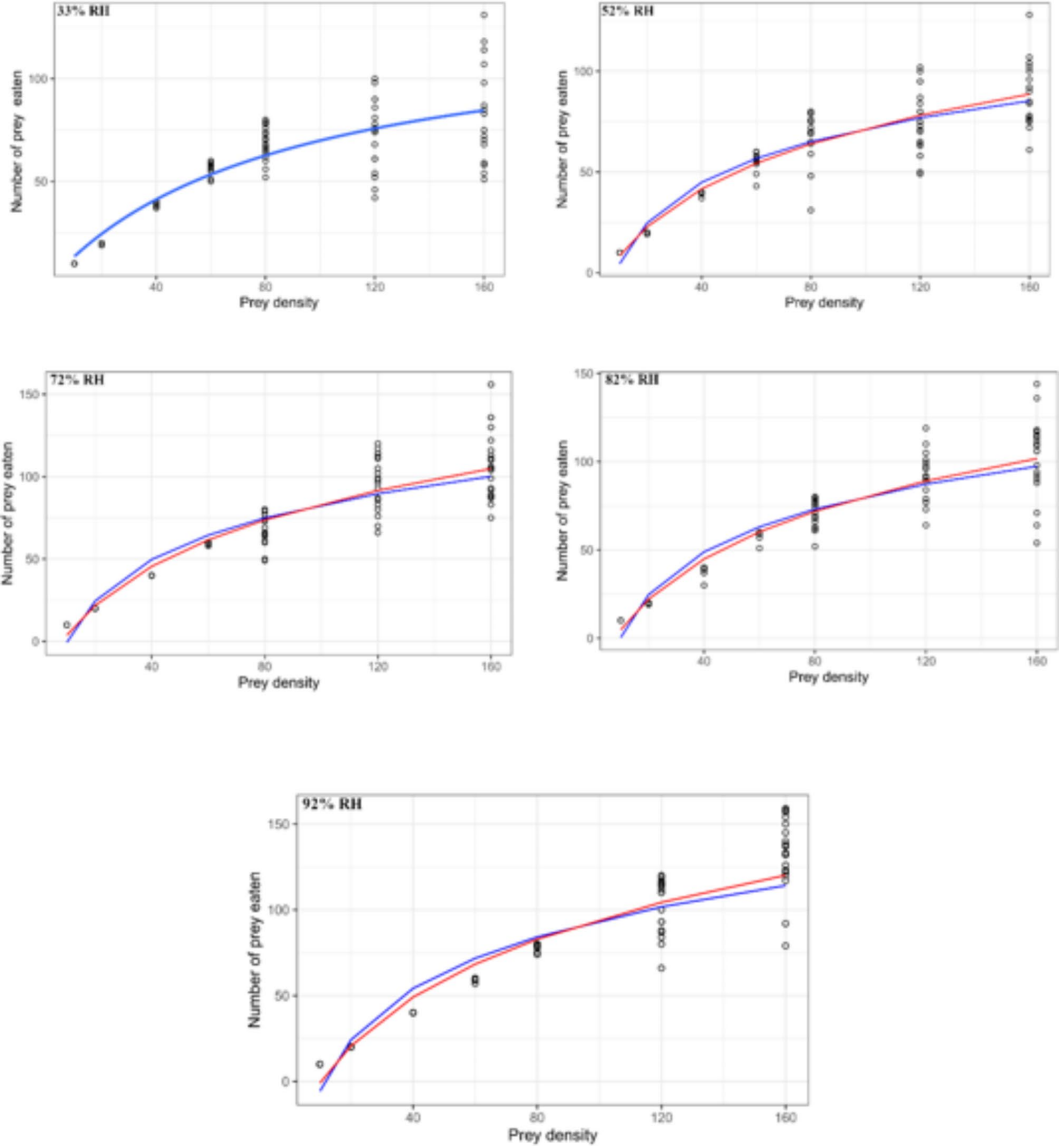
Functional responses of *Blattisocius mali* to the *Tyrophagus putrescentiae* eggs at five levels of humidity predicted from the model. Blue and red lines were drawn based on the models proposed by Hassell^12^ and Cabello et al.^24^, respectively.

The shape of the functional response curve exhibited a hyperbolic pattern at a humidity level of 33%, indicating a Type II functional response. However, at higher humidity levels, the curve displayed a nearly sigmoid shape, indicating a Type III functional response (Fig. 2). The functional response curves were drawn based on the models proposed by Hassell^12^ at 33% humidity, and both Hassell^12^ and Cabello et al.^24^ at higher humidities. The curve drawn based on Hassell’s^12^ model showed that the number of eggs eaten increased with increasing egg densities hyperbolically at 33% humidity. Whereas, the curves drawn based on models suggested by Hassell^12^ and Cabello et al.^24^ showed similar results at other humidity levels, i.e., the number of eggs eaten increased with increasing egg densities following the sigmoid shape (Fig. 3).

Estimates of functional response parameters, determined from the model proposed by Hassell^12^, revealed that *B. mali* exhibited longer handling times at lower humidities as compared to higher humidities. The handling time was significantly longer at 33% and 52% humidities but decreased with an increase in humidity up to 92% humidity (Table 3). The instantaneous attack rate (a) for Type II was 1.51 day^− 1^ at 33% humidity and the Functional Response Ratio [FRR^52^) was 196.5396 day^− 2^. The predation rate of *B. mali* was significantly affected by humidity levels (χ^2^ = 38.18; *df* = 4; *P* < 0.01) and increased with increasing humidity levels (Table 3). The parameters estimated from the model suggested by Cabello et al.^24^ showed that *B. mali* exhibited a higher potential for prey mortality and shorter handling time at higher humidities as compared to lower humidity levels. The potential for prey mortality was the highest at 92%, followed by 82%, 72%, and 52% humidity levels (Table 4). Conversely, the handling time was the lowest at 92% humidity and increased with the declining humidity up to 52%. Humidity significantly affected both the FRRs^52^ (χ^2^ = 35.231; *df* = 3; *P* < 0.01) and the predation rate (χ^2^ = 42.045; *df* = 3; *P* < 0.001), which increased clearly with increasing the humidity levels (Table 4).

**Table 1:** Results of the logistic regression analysis of the proportion of *Tyrophagus putrescentiae* eggs eaten by *Blattisocius mali* relative to the initial number of eggs provided at five humidity levels.

Humidity (%)	Coefficient	Estimate	Standard Error	*Pr* *P* (z) value
33	Constant	− 7.0050	0.8899	< 0.001
	Linear	0.7219	0.0272	0.008
	Quadratic	0.0328	0.0003	0.009
	Cubic	0.0009	0.0000	0.241
52	Constant	− 10.1200	1.2290	< 0.001
	Linear	0.1737	0.0365	< 0.001
	Quadratic	− 0.0010	0.0003	0.002
	Cubic	0.0000	0.0000	0.044
72	Constant	− 48.7600	6.5560	< 0.001
	Linear	1.2580	0.1790	< 0.001
	Quadratic	− 0.0108	0.0015	< 0.001
	Cubic	0.0030	0.0004	< 0.001
82	Constant	− 7.4890	1.0260	< 0.001
	Linear	0.0890	0.0315	0.004
	Quadratic	− 0.0034	0.0003	0.025
	Cubic	0.0004	0.0091	0.674
92	Constant	− 9.0140	3.0850	< 0.001
	Linear	0.0279	0.0902	0.756
	Quadratic	− 0.0071	0.0008	0.389
	Cubic	0.0003	0.0002	0.121

**Table 2:** Estimates of various parameters of the Real’s^51^ model, a (attack rate), T_h_ (handling time), q (scaling component), for the proportion of *Tyrophagus putrescentiae* eggs eaten by *Blattisocius mali* relative to the initial number of eggs provided at five humidity levels.

Humidity (%)	Parameter	Estimate	Standard Error	*Pr* *P* (z) value
33	a	0.2477	0.1530	< 0.001
	T_h_	0.0107	0.0007	< 0.001
	q	0.0757	0.1010	0.454
52	a	0.4320	0.2091	< 0.001
	T_h_	0.0092	0.0007	< 0.001
	q	0.3621	0.1423	< 0.001
72	a	0.5976	0.2610	< 0.001
	T_h_	0.0068	0.0007	< 0.001
	q	0.2157	0.0257	0.008
82	a	0.4460	0.2003	< 0.001
	T_h_	0.0057	0.0008	< 0.001
	q	0.3146	0.0285	0.015
92	a	0.8618	0.3021	< 0.001
	T_h_	0.0026	0.0008	< 0.001
	q	0.5522	0.0808	0.002

**Table 3:** The attack rate (a) (± SE), handling time (T_h_) (± SE), constants, b (± SE), and c (± SE), functional response ratio (FRR) (a/T_h_), and maximum predation rate (T/T_h_) of *Blattisocius mali* preying on *Tyrophagus putrescentiae* eggs under five levels of humidity, resulting from Hassell^12^ model.

Humidity (%)	a (day^− 1^)	T_h_ (day)	b	c	a/ T_h_ (Day^− 2^)	T/T_h_
33	1.5102 ± 0.11688	0.0076 ± 0.00053a (0.0071–0.0079)	–	–	196.5396	130.1406c
52	–	0.0073 ± 0.00002a (0.0069–0.0071)	0.1734 ± 0.00937	0.0981 ± 0.00129	–	136.1637c
72	–	0.0049 ± 0.00027b (0.0042–0.0052)	0.1560 ± 0.07111	0.1002 ± 0.00892	–	202.9221b
82	–	0.0043 ± 0.00028b (0.0042–0.0046)	0.3267 ± 0.00781	0.2490 ± 0.00038	–	231.5672b
92	–	0.0030 ± 0.00013c (0.0028–0.0034)	0.1485 (± 0.00377)	0.1056 ± 0.00057	–	332.8452a

Different letters within a column indicate significant differences between humidity levels (*P* < 0.05) based on 95% CI in the case of handling time and the Dunn test with Bonferroni correction in the case of maximum predation rate.

**Table 4:** The potential of mortality (α) (± SE), handling time (T_h_) (± SE), functional response ratio (FRR) (α/T_h_), and maximum predation rate (T/T_h_) of *Blattisocius mali* preying on *Tyrophagus putrescentiae* eggs under five levels of humidity, resulting from Cabello et al.^24^ model.

Humidity (%)	α	T_h_ (Day)	α/ T_h_ (Day^− 1^)	T/T_h_
33	–	-	–	–
52	0.0146 ± 0.00261c (0.0123-0.0189)	0.0066 ± 0.0009a (0.0062-0.0071)	2.2164c	151.0574c
72	0.0245 ± 0.00271b (0.0221-0.2891)	0.0055 ± 0.00056b (0.0053-0.0058)	4.3907b	178.9421b
82	0.0272 ± 0.00245b (0.2645-0.3191)	0.0053 ± 0.00038b (0.0051-0.0057)	5.0695b	185.8114b
92	0.0592 ± 0.00945a (0.0543-0.0634)	0.0046 ± 0.00023c (0.0041-0.0050)	12.7733a	215.6474a

Different lowercase letters within a column indicate significant differences between humidity levels (*P* < 0.05) based on 95% CI in the case of potential of prey mortality and handling time and the Dunn test with Bonferroni corrections in the case of FRR and maximum predation rate.

## Discussion

The results of this study showed that humidity had a significant impact on the functional response of *B. mali* when preying on *T. putrescentiae* eggs. When tested across a range of humidity levels from 33% to 92% RH, the predatory females consistently displayed a Type III response, except at the lowest humidity level of 33% where they exhibited a Type II response. At this low humidity level, the predation rate of the mite was the lowest for most prey densities. Additionally, the handling time was significantly longer at 33% and 52% humidity levels compared to higher levels of humidity. Conversely, the potential for prey mortality was much lower at 52% humidity compared to higher humidities. As the humidity increased further, the handling time decreased, reaching its shortest value at 92%. Furthermore, at this particular humidity level, the potential for mortality of *T. putrescentiae* eggs was the highest for this predatory mite.

Edaphic predatory mites have been observed to exhibit different responses to varying humidity levels to ensure their survival and growth^49,50^. Although humidity has a direct effect on the survival of soil predatory mites, there is no report on the effect of humidity on the foraging behaviour including the functional response of soil mites. To the best of our knowledge, this paper represents the first report on the effect of humidity on the predation of the soil mite.

According to previous studies, predatory mites predominantly have displayed either Type II or Type III functional responses^19^. However, there has been limited investigation into the functional responses of predatory soil mites, unlike the well-studied plant-dwelling phytoseiids. The soil mites, *B. tarsalis* and *Macrocheles robustulus* Berlese (Mesostigmata: Macrochelidae) exhibited Type II response when exposed to eggs of the potato tuber moth (PTM), *Phthorimaea operculella* Zeller (Lepidoptera: Gelechiidae)^43^. Similarly, *B. tarsalis* showed Type II response to eggs of the Guatemalan potato tuber moth *Tecia solanivora* Povolny (Lepidoptera: Gelechiidae)^53^; *Stratiolaelaps scimitus* Womersley (Mesostigmata: Laelapidae) exhibited Type II to pupae of the western flower thrips *Frankliniella occidentalis* Pergande (Thysanoptera: Thripidae)^54^ while *S. scimitus* and *Macrocheles mammifer* Berlese (Acari: Macrochelidae) to pupae of the Asian bean thrips *Megalurothrips usitatus* Bagnall (Thysanoptera: Thripidae)^55^. The macrochelid mite *Macrocheles muscaedomesticae* Scopoli (Acari: Macrochelidae) exhibited Type III functional response at 27 °C and Type II at 33 °C when exposed to varying densities of eggs of the house flies *Musca domestica* L. (Diptera: Muscidae)^31^.

Type II and Type III functional responses differ significantly about the stability of the predator–prey system. In the case of Type II, prey density is reduced in the following generations and finally becomes extinct due to negatively density-dependent predation. By contrast, in the sigmoid response, predation is initially positively density-dependent up to some threshold which may contribute to the stability of predator-prey system^23,56^. Invertebrate predators could exhibit different types of functional responses based on various factors, including temperature, prey and predator types, predator age, voracity, hunger level, etc^26–28^. As shown by the study of Daugaard et al.^26^ on climate warming effects in ciliates, an increase in temperature could lead to the shift from Type III to Type II functional response, resulting in destabilization of the predator-prey system. Contrarily, the results of our research suggested that low humidity levels could potentially destabilize the interaction between *B. mali* and *T. putrescentiae*. Several reports indicated that *B. mali* might inhabit environments with relatively high humidity. For example, in Finland, this predator was carried on the body of several species of fruit flies of the genus *Drosophila*, which inhabited moist riverside habitats^57^. Moreover, in grain or dried fruit storage rooms, *B. mali* often co-occurred with acarid mites, such as *T. putrescentiae*,* Acarus siro* Latreille, *Carpoglyphus lactis* Linnaeus, and *Glycyphagus destructor* Schrank^58–61^. Acarid mites are generally hygrophilous and susceptible to low relative humidity due to their weakly sclerotized cuticles that favour water loss through the body surface. The optimal humidity for the survival of *T. putrescentiae* lies between 80% and 90% RH at 25 ºC^62^, which also coincides with the highest probability for prey mortality and the shortest handling time of the *T. putrescentiae* eggs by *B. mali*, which was observed in this study. In contrast, the lowest relative humidity at which *T. putrescentiae* development was possible was found to be 65% at temperatures ranging from 15 ºC to 25 ºC^63^. Also in *B. mali*, the functional response parameters gradually deteriorated as the humidity decreased until reaching 52%. However, only at 33% relative humidity, there was a significant decrease in the number of eggs eaten by a predator for most prey densities, which might be related to a significant reduction of its activity to conserve energy and reduce water deficit.

Arthropods can cope with the risk of dehydration in different ways. The adaptations can be morphological (body size, integument composition), physiological (e.g. development time, respiration rate), or behavioural (e.g. aggregating, quiescence, free water uptake; increased prey consumption, transfer to sites with lower saturation deficit)^7,8^. A decrease in the activity of *B. mali* females and their foraging in low humidity could have a significant adaptive value due to the limited availability of their prey under such conditions. However, studies have shown that both male and female mould mites can survive short drops in relative humidity to 15% for 24–48 h (such as during migration). If they are unable to escape or find moisture for longer periods, both the mites and their eggs will become desiccated, as demonstrated by Eaton and Kells^63^.

As the previous studies showed, phytoseiids may behave completely differently from *B. mali*. Mori and Chant^16^ studied the functional response of *Phytoseiulus persimilis* Athias-Henriot (Mesostigmata: Phytoseiidae) to mixed stages of the two-spotted spider mite, *Tetranychus urticae* Koch (Trombidiformes: Tetranychidae), at 50%, 70%, and 100% RH while Döker et al.^64^ examined *Neoseiulus californicus* (McGregor) (Mesostigmata: Phytoseiidae) about the eggs of *T. urticae*, in a humidity range of 30–90%RH similar to that in our study. Both phytoseiid species are associated with *Tetranychus* species. *Phytoseiulus persimilis* is a specialized predator of *Tetranychus* species while *N. californicus* is a selective predator of tetranychid mites and well adapted to heavy webbing found within their colonies^65^. *Phytoseiulus persimilis* showed generally a less common ‘domed’ functional response^16^ while *N. californicus* displayed a Type II response^64^. Interestingly, in both species, a decrease in humidity led to an increase in prey consumption. In *P. persimilis*, the highest prey consumption was at 30% RH, while in *N. californicus* the attack rate was the highest at 50% RH, which has been explained by the authors of both studies as adaptation aimed at preventing water loss. Conversely, at very high humidity levels, the activity of phytoseiids decreased, leading to reduced prey consumption. As in the case of *B. mali*, the predation rate depended on the potential availability of prey. Contrary to mold mites, high humidity inhibited the development of spider mite populations, while low humidity favoured their increased growth rate^65^.

Predators exhibiting Type III response are especially promising biological control agents due to their ability to consume prey in a density-dependent manner, which may stabilize the prey-predator interaction, and regulate the population of prey species^67,68^. Similar hopes were raised by *B. mali*, which in this study, exhibited Type III functional response to increasing densities of *T. putrescentiae* eggs within relatively a broad range of humidity favouring the development of the prey. The estimates of parameters of functional response by both Hassell^12^ and Cabello et al.^24^ models showed similar trends across different levels of humidity. It confirmed the usefulness of the model by Cabello et al.^24^ and the parameter α, i.e. potential of mortality, in further research on the behaviour of predators exhibiting Type III functional response. The instantaneous attack rate, a, is a parameter characterizing Type I, and together with handling time, it also describes Type II functional response^12^. However, in the sigmoid response, apart from handling time there are also two model parameters, b, and c, that are constants and have no biological meaning. Instead, the potential of mortality, proposed by Cabello et al.^24^ for Type III, is the affinity of a predator at which a predator causes the mortality of a prey. While a high attack rate means that the predator is adept at quickly removing prey from the areas where it is foraging, a high potential of mortality means that the predator has a very high degree of efficacy, causing high mortality of prey^24^. In our findings, the shortest handling time and the highest potential of mortality were observed at 92% humidity which was not only optimal humidity for mould mites but also seemed to be optimal for *B. mali* foraging. However, when the two parameters were amalgamated in the novel FRR (i.e. a/ T_h_ or α/T_h_), which considers the joint effects of the attack rate or potential of mortality and handling time parameters^52^, we found a clear and significant increase in FRR over increasing humidity levels. It is more important that under suitable conditions of food, temperature, and humidity, eggs make up nearly 50% of the population of *T. putrescentiae*^69^.

Although our study suggests a high potential of the predator to reduce the population of *T. putrescentiae* at higher humidities, research is necessary on other prey stages, which behaviour, e.g., emission of alarm pheromones, clumping to reduce water loss, or burrowing in food to limit respiration^62,63,70^, may substantially hinder predation and also influence both functional response and its parameters. While the findings provide valuable insights into the potential efficacy of *B. mali* against *T. putrescentiae*, the scope of the study may limit its ability to fully assess its effectiveness under Real-world conditions. Future studies are needed to determine the numerical response, interference, and efficiency of converting ingested food into egg biomass (ECI) to gain a more thorough understanding of its effectiveness as a biocontrol agent against mould mites. Moreover, further tests should be performed to confirm that the shift from Type III to Type II functional response at low humidity may destabilize the system between *B. mali* and *T. putrescentiae* or other prey types.

## Methods

### Mites

The initial culture of *T. putrescentiae* reared on instant dry bakers’ yeast and wheat bran (50/50% by weight), was taken from the mass rearing of the Department of Plant Protection, Warsaw University of Life Sciences, Warsaw, Poland. *Tyrophagus putrescentiae* adults were chosen and reared using the same quantity of yeast and wheat bran (50/50% by weight) in glass Petri dishes (90 mm in diameter) for obtaining 24-h eggs following the method developed by Pirayeshfar et al.^71^. Petri dishes were placed on top of water-saturated foam located inside larger plastic containers (120 mm diameter, 200 mm high), which were half-filled with water and covered with a lid containing small pores for ventilation. The foam was covered with wet tissue paper to prevent the mites from escaping^35^. The cultures were maintained in a Sanyo Environmental Test Chamber (Panasonic MLR-350) in darkness, at 26 °C and 95 ± 5% RH.

The culture of *B. mali* was maintained on various stages of *T. putrescentiae* in wheat bran in the laboratory of the Department of Plant Protection at Warsaw University of Life Sciences, Warsaw, Poland^47,62,63^. The species of predatory mite was previously identified morphologically and confirmed molecularly by DNA barcoding^45,72^. The rearing unit consisted of soaked foam platforms (220 mm × 150 mm × 25 mm), which were covered with foil and placed within broader vessels filled up with water. The cultures of *B. mali* were maintained in a Sanyo Environmental Test Chamber (Panasonic MLR-352-PE), at 23 °C, with a photoperiod of 16/8 h (L/D) and a humidity of 85 ± 5%.

### Experimental set-up

The experimental unit consisted of a Plexi-glass cage (38 mm × 30 mm × 4 mm), in which a round-shaped hole (8 mm) was drilled. A piece of white filter paper was attached to the lower surface of the cell and a suitable glass coverslip (18 mm × 18 mm) was placed on its upper surface using paraffin wax to prevent the escape of predatory mites. Before 24 h of choosing the female predator, the colony was always fed with sufficient amount of the mixed life stages of *T. putrescentiae* reared on yeast. The well-fed female predator was randomly chosen from the colony and exposed to seven densities, 10, 20, 40, 60, 80, 120, or 160 of 24-h *T. putrescentiae* eggs at five different levels of humidity, 33%, 52%, 72%, 82%, and 92%, and at a constant temperature of 25 °C and photoperiod of 16 L:8D h in a cooled incubator (MIR-154-PE) for 24 h. The humidity levels of 33%, 52%, 72%, 82%, and 92% were maintained by the solutions of MgCl_2_, Mg (NO_3_)_2_, NaCl, KCl, and KNO_3_, respectively in a desiccator^73^. The eggs of *T. putrescentiae* were separated from other stages by sieving the rearing colonies through a 100 μm mesh screen^74^. The eggs were transferred to the chamber/cell of the cage using a fine paintbrush and were scattered evenly on the base of the chamber at all densities. After 24 h, the predators were removed and the number of eggs eaten was counted by excluding the remained eggs. Cages from which a live *B. mali* was not recovered, because of loss or death, were not included in the analysis. Each egg density was replicated twenty times at each humidity level.

### Functional response

To analyze the effect of humidity and density of prey on the consumption of the eggs of *T. putrescentiae* by *B. mali*, Generalized Linear Models (GLM) with Poisson probability distribution were applied. As a post hoc test, we used Tukey’s linear contrast.

The functional response data were analyzed in two stages. First, we determined the type of functional response and then estimated the parameters of the functional response. The type of the functional response was determined by, logistic regression of the proportion of prey killed as a function of initial density^72^ and the generalized functional response equation of Real^41^. The polynomial logistic regression equation with binomial distribution (Eq. 1) to determine the type of functional response was fitted as under:

1 $$\:\frac{{N}_{a}}{{N}_{0}}=\:\frac{\text{e}\text{x}\text{p}({P}_{0}+{P}_{1}{N}_{0}+{P}_{2}{N}_{0}^{2}+{P}_{3}{N}_{0}^{3})}{1+\:\text{e}\text{x}\text{p}({P}_{0}+{P}_{1}{N}_{0}+{P}_{2}{N}_{0}^{2}+{P}_{3}{N}_{0}^{3})\:}$$

where $\:\frac{{N}_{a}}{{N}_{0}}$ is the proportion of prey eaten, N_a_ is the number of prey eaten, N_0_ is the initial number of prey density offered, $\:{P}_{0}$ is the intercept, $\:{P}_{1}$, $\:{P}_{2}$, and $\:{P}_{3}$ are the linear, quadratic, and cubic coefficients, respectively. The coefficients were estimated using the maximum likelihood method. The values of linear and quadratic coefficients indicate the type of functional response. Type I responses are described by an intercept or constant positive slope ($\:{P}_{0}$). In the Type II responses, the linear coefficient (P_1_) $\:{P}_{0}$ is negative and the proportion of prey eaten declines monotonically with the initial number of prey offered. Whereas, the linear coefficient (P_1_) $\:{P}_{0}$ is positive and the quadratic coefficient ($\:{P}_{2})$ is negative in Type III responses, which are characterized by an increase in the proportion of prey eaten with the prey density offered up to an inflection point and then decreases^74^.

The modified Holling disc equation proposed by Real^51^ (Eq. 2) was as under^75^:

2 $$\:\:{N}_{a}=\:\frac{aT{N}_{0}^{(q+1)}}{1+a{T}_{h}{N}_{0}^{(q+1)}}$$

where N_a_ is the number of prey eaten, N_0_ is the initial number of prey densities offered, a is the predator’s instantaneous attack rate or searching efficiency (The rate of successful search), T_h_ is the handling time (Time spent by the predator in subduing, pursuing, eating, and digesting the prey), T is the time length of the assay, and q is the scaling component that determines the shape of the curve. The functional response can be a Type I (linear, q = 0 and T_h_ = 0), Type II (hyperbolic curve, q = 0, T_h_ > 0), or a Type III (sigmoid curve, q > 0, T_h_ > 0).

After determining the correct shape of the functional response, the functional response parameters, i.e., instantaneous attack rate (a), handling time (T_h_), and the potential for prey mortality (α), were estimated after fitting to proper models. Data were fitted to the equations proposed by Hassell^12^ (Eqs. 3 and 4) and Cabello et al.^24^ (Eq. 5), using non-linear least square regression, as the depleted preys were not replaced throughout the experiment:

3 $$\:\text{H}\text{a}\text{s}\text{s}\text{e}\text{l}\text{l}\:\text{T}\text{y}\text{p}\text{e}\:\text{I}\text{I}:\:{N}_{a}=\:{N}_{0}\:[1-\text{exp}\left\{-aP\left(T-\frac{{T}_{h}{N}_{a}}{P}\right)\right\}]$$

4 $${\text{Hassell Type III:}}\:\:{N_a} = \:{N_0}\:[1 - {\text{exp}}\left\{ { - \frac{{b{N_0}}}{{1\: + c{N_0}}}\left( {T - \frac{{{T_h}{N_a}}}{P}} \right)} \right\}$$

5 $$\:\text{C}\text{a}\text{b}\text{e}\text{l}\text{l}\text{o}\:et\:al.\:\text{T}\text{y}\text{p}\text{e}\:\text{I}\text{I}\text{I}:\:{N}_{a}=\:{N}_{0}\:[1-\text{exp}\left\{-\frac{\alpha\:{N}_{0}}{1\:+{T}_{h\:}(\text{exp}\left(-\alpha\:\right)-1){T}_{h}}\left(T-\frac{{T}_{h}{N}_{a}}{P}\right)\right\}]$$

where N_a_ is the number of prey eaten, N_0_ is the initial number of prey density offered, a is the predator’s instantaneous attack rate, T_h_ is the handling time, P is the number of predators used, T is the time length of the assay, α is the potential of mortality of the predator, and b and c are the constants that relate a and N_0_ in Type III functional response as a= $\:\frac{b{N}_{0}}{1\:+c{N}_{0}}$. In our experiment, *P* = 1 and T= 1 day. The parameters, T_h_ and α among different humidities, were obtained using a non-linear least square regression procedure and were compared based on their confidence intervals (± 95% CI) obtained, i.e., if the CI does not overlap, the difference between the means is significant (*P* < 0.05)^76^. To calculate Confidence Intervals (CI), we used the permutation test described by Ernst^77^. We obtained two different kinds of functional responses at different levels of humidity. Therefore, the differences in attack rate among different humidities were not estimated, as the instantaneous attack rate was a constant in Type II functional response but it was a variable that varied with prey densities in Type III functional response^10^. To amalgamate and further compare the functional response parameters a, α, and T_h_ among humidity levels, the functional response ratio (FRR)^52^ was estimated using either the attack rate (a) or potential of prey mortality (α) divided by the handling time (T_h_). The FRR has advantages due to combining either a or α with T_h_, as high values for a or α and low values for T_h_ would result in high predatory impacts on the ecosystem. Moreover, the predation rate of the predator was estimated by dividing the duration of assay (T) by the handling time (T_h_). One-way Kruskal–Wallis’s rank sum tests were used to test whether FRRs and the predation rate differed across humidity levels. We used Dunn test as post hoc test with Bonferroni corrections for comparison. In addition, the proportion of prey eaten by the predator at different densities was analyzed using Generalized Linear Models (GLM) with gamma probability distribution. All statistical analyses were performed using R version 4.3.0 (The R Foundation for Statistical Computing, Vienna, Austria)^78^.

## Data Availability

The data used in this study are available by email request to the corresponding author (email: manoj_jena@sggw.edu.pl).
